# Supplementary material for: An insect-scale artificial visual-olfactory bionic compound eye
Source: Nat Commun. 2026 Feb 2;17:2259. doi: 10.1038/s41467-026-68940-0 (PMC12966333; doi:10.1038/s41467-026-68940-0)
Supplement: Supplementary file 1 — Supplementary Information [file 41467_2026_68940_MOESM1_ESM.pdf]

An insect-scale artificial visual-olfactory bionic compound eye

Jiachuang Wang[1,2,4]*, Shuai Wei*[1,2]*, Nan Qin*[1,2]**, and Tiger H. Tao*[1,2,3,4,5]***

Email: qinnan@mail.sim.ac.cn, tiger@mail.sim.ac.cn

[1]State Key Laboratory of Transducer Technology, Shanghai Institute of Microsystem and Information Technology, Chinese Academy of Sciences, Shanghai 200050, China

[2]School of Graduate Study, University of Chinese Academy of Sciences, Beijing 100049, China

[3]Center of Materials Science and Optoelectronics Engineering, University of Chinese Academy of Sciences, Beijing 100049, China

[4]2020 X-Lab, Shanghai Institute of Microsystem and Information Technology, Chinese Academy of Sciences, Shanghai 200050, China

[5]School of Physical Science and Technology, ShanghaiTech University, Shanghai 200031, China

Keywords: Artificial Compound Eye, photodetector array, micro-lens array, visual-olfactory fusion.

Table of contents:

Supplementary Note 1. Detailed fabrication process of bionic compound eye (bio-CE).

Supplementary Note 2. Lens-photodetector alignment fabrication process.

Supplementary Note 3. Mathematical model of spatial object detection based on cylindrical compound eye structure.

Supplementary Figure 1. Schematic diagram of the bio-CE fabrication process.

Supplementary Figure 2. Lens-photodetector alignment fabrication process based on two-photon polymerization.

Supplementary Figure 3. Cylindrical compound eye structure model fitting and activation form.

Supplementary Figure 4. Flicker effect in bio-CE imaging.

Supplementary Figure 5. Supplement Characterization of different types of micro-lens array.

Supplementary Figure 6. Normalized intensity distributions along the X-axis and Y-axis.

Supplementary Figure 7. Object attribute recognition experiment.

Supplementary Figure 8. The bio-CE imaging model.

Supplementary Figure 9. The manufacturing details and layout design of the flexible electrode array.

Supplementary Figure 10. The derivation process of filling coefficient factor.

Supplementary Figure 11. Multilayer composite film (PDMS-SU8-Polyimide) bending simulation and effect on light response speed.

Supplementary Figure 12. Absorption spectra of photoelectric materials.

Supplementary Figure 13. Morphology characterization of P3HT/PCBM/PbS QDs organic thin film.

Supplementary Figure 14. Flicker frequency response characteristics of bio-CE.

Supplementary Figure 15. Optical response uniformity of all pixels.

Supplementary Figure 16. Stability of photo response under 10000 cycles.

Supplementary Figure 17. Lifetime of bio-CE after PDMS encapsulation.

Supplementary Figure 18. Details and connections of the bio-CE based unmanned vehicle platform.

Supplementary Figure 19. Alignment printing process and results.

Supplementary Figure 20. Fixed curvature holder of bio-CE.

Supplementary Figure 21. Simulation of biomimetic micro lens array ray tracing.

Supplementary Figure 22. Resolution of the fabricated microlens array.

Supplementary Figure 23. Imaging of different shapes and movements by bio-CE.

Supplementary Figure 24. Test results of bio-CE microsystem in dynamic environments with multiple moving obstacles.

Supplementary Figure 25. Colorimetric response under different concentration.

Supplementary Figure 26. Humidity stability of colorimetric array.

Supplementary Figure 27: The lens receiving angle and the cutting ability of edge rays.

Supplementary Table 1. Comparison of design parameters and performance of artificial compound eyes.

Supplementary Table 2. Comparison of the performance of bio-CE with different types of biological eyes.

Supplementary Table 3. Material parameters for flexible electrode bending simulation.

Supplementary Table 4. The critical concentration and basic properties of target gases.

Supplementary Note 1. Detailed fabrication process of bionic compound eye (bio-CE).

Aluminum sacrifice layer:

1. Clean Si wafer (acetone, isopropyl alcohol (IPA), deionized (DI) water).
2. Sputter 1000 nm of Aluminum by sputter coater.

Polyimide substrate:

3. Spin coat with polyimide (PI, poly (pyromellitic dianhydride-co-4,4'-oxydianiline) 2000 rpm, 30 sec.
4. Anneal at 110°C for 3 min and 150°C for 10 min.
5. Anneal at 325°C for 60 min in N_2 atmosphere.
6. Repeat step 2&3&4 twice.
7. Expose to oxygen plasma (208 W) for 5 min.

Bottom via electrodes:

8. Pattern photoresist (PR; AZ5214, 3000 rpm, 30 sec) with optical lithography (Mask #1: Metal 1).
9. Expose to oxygen plasma (105 W) for 1 min.
10. Deposit 10/150 nm of Cr/Au by metal evaporator.
11. Lift-off PR by acetone.
12. Clean the processed wafer (ethanol, DI water).

Dielectric layer:

13. 120°C pre-bake wafer for 20 min.
14. Pattern SU8 (SU8-2005, 3000 rpm, 30 sec) with optical lithography (Mask #2: Dielectric Layer).
15. Bake patterned SU8 at 165 °C for 90 min.

Metal Addon:

16. Pattern PR with optical lithography (Mask #3: Metal 2).
17. Expose to oxygen plasma (105 W) for 1 min.
18. Deposit 20/350 nm of Cr/Au by metal evaporator.
19. Lift-off PR by acetone.
20. Clean the processed wafer (ethanol, DI water).

Interdigital electrode:

21. Pattern PR with optical lithography (Mask #4: Metal 3).
22. Expose to oxygen plasma (105 W) for 1 min.
23. Deposit 10/100 nm of Cr/Au by metal evaporator.
24. Lift-off PR by acetone.
25. Clean the processed wafer (ethanol, DI water).

SU8 encapsulation:

26. 120°C pre-bake wafer for 20 min.
27. Pattern SU8 with optical lithography (Mask #5: Encapsulation).
28. Bake patterned SU8 at 165 °C for 90 min.

Aluminum hard mask:

29. Sputter 500 nm of Aluminum by sputter coater.
30. Pattern PR (LC100A, 1000 rpm, 30 sec) with optical lithography (Mask #6: Hard Mask).
31. Anneal at 120°C for 30 min.
32. Immerse processed mask in aluminum corrosive acid for 4 min.
33. Clean the processed wafer (DI water).

Etching release window:

34. Expose to oxygen plasma (208W) for 2 min.
35. Repeat step 34 5~6 times, etch release window (SU8/PI) to sacrifice layer (Al).
36. Clean the processed wafer (DI water).

Device release:

37. Etch Al sacrifice layer by HF for 5 min.
38. Clean the released device (DI water) and fixed on a clean wafer.

Semiconductor layer fabrication and PDMS encapsulation:

39. 20 mg of [6,6]-Phenyl C61 butyric acid methyl ester (PC60BM, 160848-22-6, Aladdin), 20 mg Poly(3-hexylthiophene-2,5-diyl) (P3HT, 104934-50-1, Aladdin) and 5 mg PbS quantum dot (Aladdin) were dissolved in 1 mL of anhydrous chlorobenzene (Aladdin) in order and let stirring overnight (20 hours) at 70 °C in a glove box under nitrogen atmosphere.

40. Spin coat with as prepared P3HT/PCBM/PbS QDs solution (2500 rpm, 30 s) in N2 atmosphere.

41. Anneal at 120°C for 45 min.

42. Cut the PDMS thin film (50 μm thickness) into a suitable size and soak in 2-Acetoxy-1-methoxypropane (PGMEA) and IPA for 30 min and 20min, respectively.

43. Expose the PDMS film to ultraviolet induced ozone for 3 min to remove organic residue.

44. Cover the Photoactive region with the preprocessed PDMS film on a 95 °C hotplate.

45. Place the device in a vacuum dryer for 12 hours before lens fabrication.

Lens fabrication:

46. Dip PR (IP-DIP, Nanoscribe) onto the PDMS encapsulation layer.

47. Measure the height difference between PDMS-PR and PDMS-device by 2PP platform (Nanoscribe, GT2 Professional).

48. Adjust lens design accordingly and load on 2PP platform.

49. Align printing coordinate with substrate coordinate, proceed polymerization.

50. Develop in PGMEA (15 min) and IPA (5 min).

51. Detach device from silicon wafer.

The corresponding manufacturing process diagram and layout design can refer to Supplementary Figure 1 and Supplementary Figure 9.

Supplementary Note 2. Lens-photodetector alignment fabrication process.

In this supplementary note, we conducted a detailed explanation of the alignment manufacturing scheme between the micro-lens array and the photoelectric detector array, and used schematic diagrams (Supplementary Figure 2) to display the design details in each step.

Correction of coordinate systems:

Accurate alignment of the multi-level coordinate systems is essential for ensuring precise micro-scale fabrication. Prior to structural printing, we performed a comprehensive calibration procedure to synchronize the galvanometer, stage, and writing area coordinate systems according to the manufacturer's established protocol. The calibration process commenced with the printing of a fine cross-marker on a prepared silicon substrate (Supplementary Figure 2A), whose geometric center was designated as the origin of the galvanometer coordinate system. This marker serves as a critical reference point for all subsequent alignment steps.

The calibration protocol continued with the adjustment of the laser direct-writing area's reference frame. Using the software interface, we dynamically manipulated the reference grid by dragging and zooming operations until perfect alignment was achieved with the pre-printed cross-marker (Supplementary Figure 2B). This crucial step established spatial correspondence between the virtual writing coordinates and physical substrate positions, effectively registering the origin within the writing zone. The alignment precision was verified through stage movements and real-time image processing, ensuring coordinate consistency across all systems.

To validate the calibration accuracy, we employed a functional verification approach by printing a single microlens structure at the defined coordinate origin (Supplementary Figure 2C). Subsequent microscopic inspection confirmed the lens was precisely positioned relative to the alignment marker, with a measured placement error of less than 1 μm. This quantitative validation protocol not only verified the coordinate system alignment but also confirmed the system's readiness for complex multi-structure fabrication. The entire calibration process, including verification, required approximately 15 minutes and was performed once per substrate loading to maintain spatial accuracy throughout the fabrication session.

Loading micro-lens array model and photodetector array installation:

Following the establishment of the coordinate origin, the STL model of the micro-lens array was imported into the fabrication software for printing file generation. To ensure precise alignment with the photodetector array on the substrate, the initial position of the lens model was designed with a crossbar marker. The complete micro-lens array was fabricated

sequentially in a snake-like patterning scheme (Supplementary Figure 2D), optimizing the writing path for efficiency and minimal stage movement.

The encapsulated photodetector array was then mounted on the printing stage, allowing clear observation of alignment markers and pixel structures within the direct-writing area (Supplementary Figure 2E). To achieve accurate registration between the lens model and underlying pixels, angular displacement correction was performed and the starting position was finalized prior to initiating the final printing process. This alignment verification ensured precise spatial correspondence between the fabricated optical elements and the photodetector pixels.

Rotating correction and alignment printing of the lens model:

The mounting process of the photodetector array on the substrate holder introduces inherent angular misalignment due to mechanical tolerances in the fixture system. This randomness prevents guaranteed orientation consistency between the pixel arrangement and the micro-lens model's printing direction, potentially causing significant optical performance degradation. To address this critical alignment challenge, we implemented a multi-point registration protocol for precise rotational correction of the micro-lens model.

The angular deviation was quantified using a set of alignment markers pre-patterned at four corners of the photodetector array (Supplementary Figures 2F, G and H). These markers form a reference coordinate system that captures the actual orientation of the detector pixels relative to the printing coordinate system. Through coordinate transformation processing (Supplementary Figure 2I), which involves calculating the centroid positions of all markers and performing singular value decomposition, the precise rotational offset was determined and automatically applied to the micro-lens model.

Following model transformation, the updated micro-lens array was re-imported into the writing software (Supplementary Figure 2J and K). The verification phase confirmed successful registration, with residual alignment errors measuring less than 1 μm across the entire printing area. This sub-pixel level accuracy ensures precise spatial correspondence between each micro-lens optical axis and the underlying photodetector pixels, which is essential for maintaining optimal light collection efficiency and cross-talk minimization in the final bio-CE device.

Micro lens array splicing printing:

The micro-lens array was fabricated using a group-wise splicing strategy, wherein 20 lenses (arranged in a 4 × 5 configuration) were aligned and printed as a single unit. This process was repeated systematically until complete coverage of all photodetector pixels was achieved. Compared to single-exposure full-array printing, the splicing approach offers significantly

improved alignment accuracy by minimizing error accumulation caused by piezoelectric stage drift over large displacements.

Furthermore, the group-wise method enables real-time process monitoring and intervention. Any processing anomalies, such as bubble formation or localized overexposure, can be immediately detected and addressed without compromising the entire array. This segmented fabrication strategy thus enhances yield and ensures consistent optical quality across the bio-inspired compound eye device.

Supplementary Note 3. Mathematical model of spatial object detection based on cylindrical compound eye structure.

In this supplementary note, we present a generalized distance detection model based on bio-CE's special imaging form. Through the derivation of geometric formulas, the mathematical relationship between the bio-CE activation form and target distance is elected. Furthermore, the geometric model provides a design template for artificial compound eyes on various scales with specific detection capabilities.

Structure parameters description:

It is worth noting that there are two types of ommatidium spacing, S_x in the direction of the bending radius of the column surface and S_y in the direction of the column axis.

Radius of curvature (R): the distance from the column section center to the pixel surface.

Lens height (h): total height of the fabricated micro-lens.

Substrate thickness (t): thickness of PDMS substrate.

Acceptance angle ($\Delta\rho$): incident angle range of the lens that can be received by photodetectors.

Interommatidial angle ($\Delta\varphi$): the angle between the center axis of the adjacent lens.

Diameter of photoreceptor (d): effective diameter which can detect the incident light.

Vertical spacing (S_y).

Horizontal spacing (S_x).

Axial direction (y).

Radius direction (x).

The activation form of bio-CE on axial and radius direction:

For the cylindrical compound eye structure, the micro-lens array is arranged in a straight line with S_y spacing in the direction of column axis (y), the pixel activation form in the plane where the target and cylindrical axis are located are shown in the Supplementary Figure 3C. Based on the basic triangular function, The activation length ($l_{active,\ y}$) and number of activated ommatidia ($n_{active,\ y}$) caused by a spot light source can be obtained by formula S3.2 and S3.3.

$$\tan\left(\frac{\Delta\rho}{2}\right)=\frac{l_{active,\ y}}{2D} \tag{S3.1}$$

$$l_{active,\ y}=2D\tan\left(\frac{\Delta\rho}{2}\right) \tag{S3.2}$$

$$n_{active,\ y} = \frac{l_{active,\ y}}{S_y} = \left[\frac{2D}{S_y}\tan\left(\frac{\Delta\rho}{2}\right)\right] \tag{S3.3}$$

On the direction of the cylindrical radius (x), the linear array of the lens is bent, and each lens towards different detection directions. The activation length ($l_{active,\ x}$) and number of activated ommatidium ($n_{active,\ x}$) on the closest row could be obtained by formula S3.6 and S3.7.

$$\frac{\sin\left(\pi - \frac{\Delta\rho}{2}\right)}{D + R + t + h} = \frac{\sin\left(\frac{\Delta\rho}{2} - \frac{\alpha_{active,\ x}}{2}\right)}{R + t + h} \tag{S3.4}$$

$$\alpha_{active,\ x} = \Delta\rho - 2\arcsin\left(\left(\frac{R + t + h}{D + R + t + h}\right) \cdot \sin\left(\frac{\Delta\rho}{2}\right)\right) \tag{S3.5}$$

$$l_{active,\ x} = \alpha_{active,\ x} \cdot \frac{\pi \cdot (R + t + h)}{180} \tag{S3.6}$$

$$n_{active,\ x} = \left[\frac{l_{active,\ x}}{S_x}\right] = \left[\frac{\alpha_{active,\ x}}{S_x} \cdot \frac{\pi \cdot (R + t + h)}{180}\right] \tag{S3.7}$$

Geometric model of the generalized activation form of bio-CE:

Furthermore, when the target position deviates from a specific x-direction plane with ΔH, the activation form in this direction can be found by a slightly complicated model. Firstly, we calculate the direct distance between the object and the origin point (OS) using the Pythagorean theorem in ΔOAS, then derive BS in ΔOBS by the Law of cosines. Finally, we calculate AB in ΔABS and get the activation length ($l_{active,\ x}$).

$$OS = \sqrt{(\Delta H)^2 + (R + t + h + D)^2} \tag{S3.8}$$

$$BS = \sqrt{(R + t + h)^2 \cdot \cos^2\left(\frac{\Delta\rho}{2}\right) - (R + t + h)^2 + (\Delta H)^2 + (R + t + h + D)^2} - (R + t + h) \cdot \cos\left(\frac{\Delta\rho}{2}\right) \tag{S3.9}$$

$$AB = \sqrt{BS^2 - \Delta H^2} \tag{S3.10}$$

$$\alpha_{active,\ x} = 2\arccos\left(\frac{(R + t + h)^2 + (R + t + h + D)^2 - AB^2}{2(R + t + h)(R + t + h + D)}\right) \tag{S3.11}$$

$$l_{active,\ x} = \alpha_{active,\ x} \cdot \frac{\pi \cdot (R + t + h)}{180} \tag{S3.12}$$

The relationship between target distance and activation form of bio-CE:

According to the aforementioned geometric model derived from the radius direction and axis direction, the correlation between the activation area of bio-CE and the target distance can be obtained through the following definite integral (S3.13):

$$S_{active} = 2\int_{0}^{\frac{l_{active,\,y}}{2}} l_{active,\,x}\, d(\Delta H) \tag{S3.13}$$

To avoid the huge computational burden of integration, we use the elliptic area formula to approximate the activation area of the cylindrical compound eye structure. As shown in formula S3.14, The major and minor axes of an ellipse are $l_{active,\,x}$ and $l_{active,\,y}$, respectively. The accuracy and rationality of this fitting method in the close range are verified in Supplementary Figure 3:

$$S_{active'} = \frac{\pi}{4} \cdot l_{active,\,x} \cdot l_{active,\,y} \tag{S3.14}$$

The calculated distance-activation area relationship is suitable for the ideal spotlight source situation. It is worth noting that the changes in the activation area are independent of the changes in the target's light intensity, which maintain a linear relationship with the target distance. For the situation of complex light sources, objects can be abstracted into a combination of spot light sources with different locations and intensities. The qualitative relationship of the distance-activated area is also applicable.

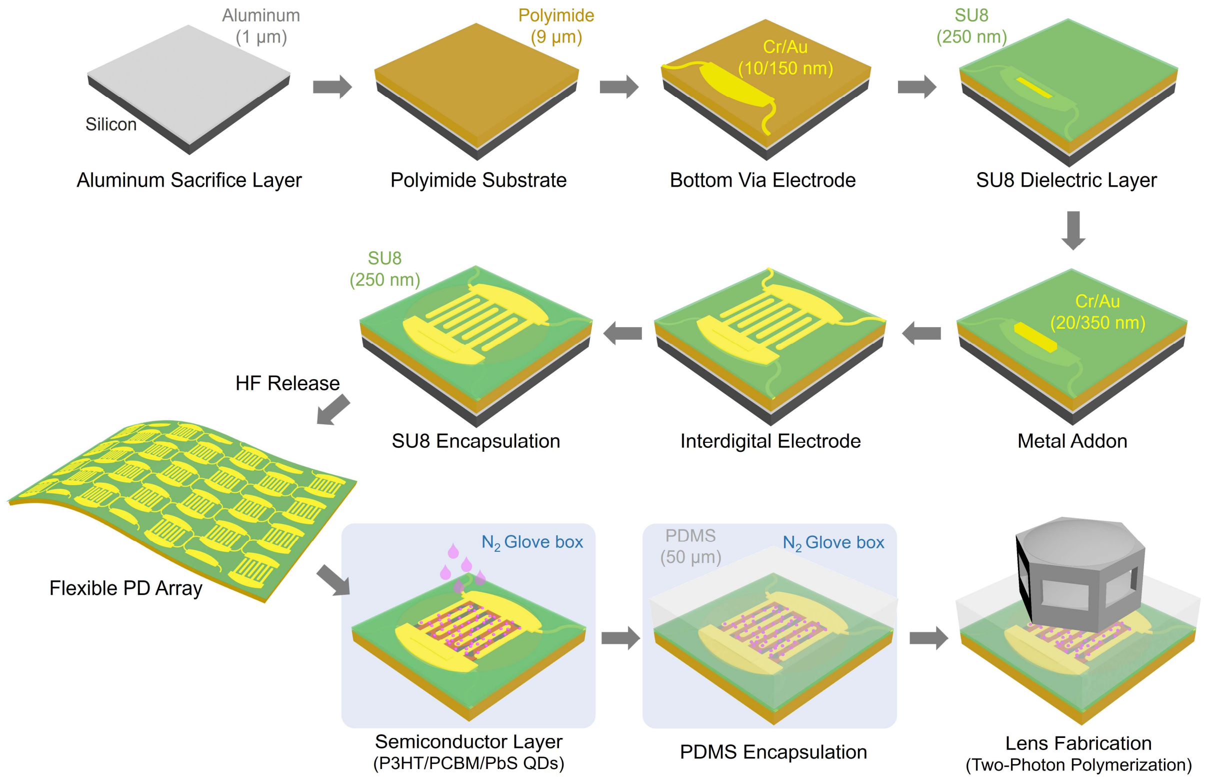

Supplementary Figure 1: Schematic diagram of the bio-CE fabrication process. The bio-CE's preparation process includes a flexible electrode array based on the 4-inch MEMS platform, P3HT/PCBM/PbS QDs organic semiconductor film preparation, and a micro-lens array based on two-photon polymerization technology. The detailed process steps are presented in Supplementary Note 1.

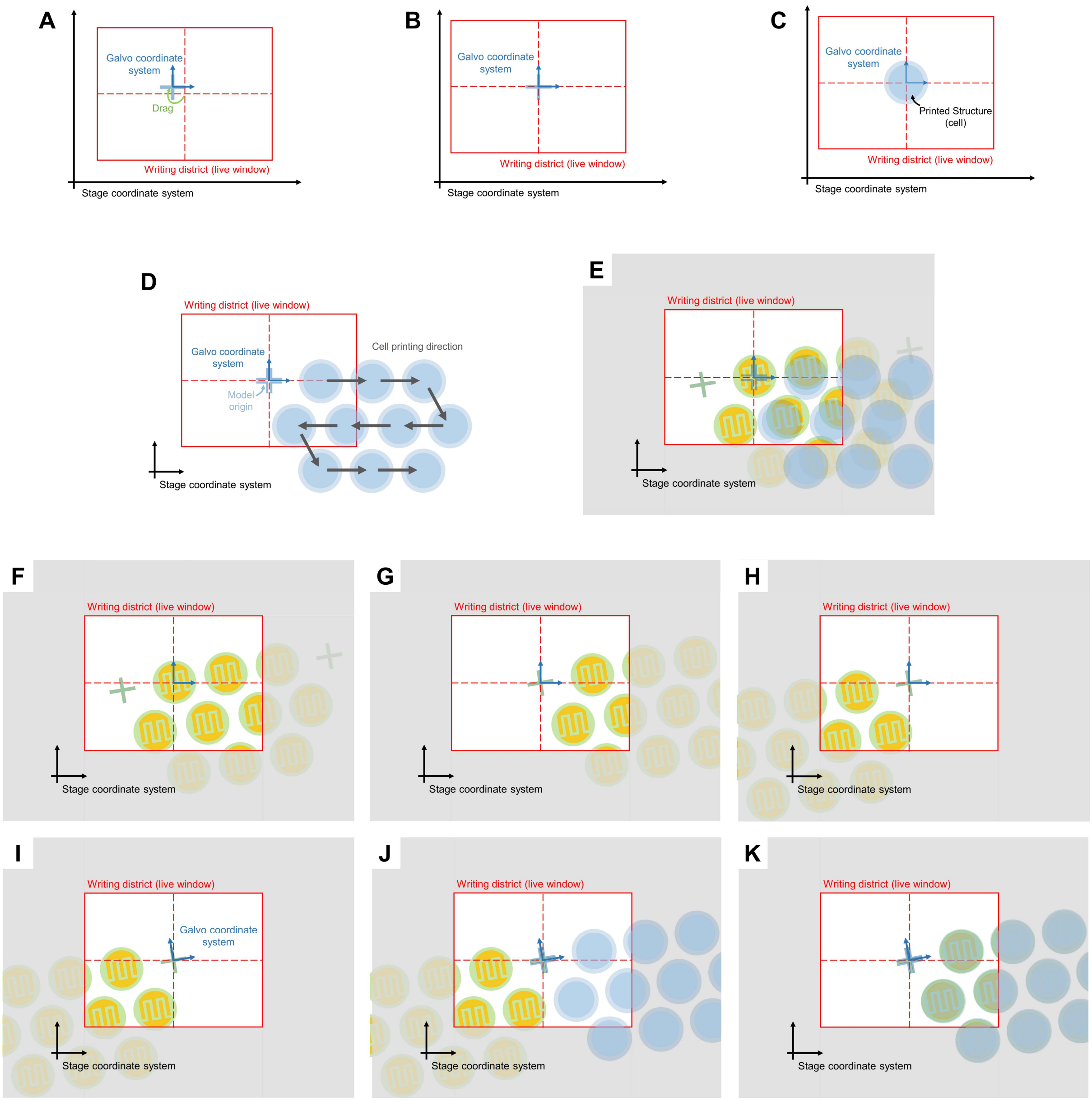

Supplementary Figure 2: Lens-photodetector alignment fabrication process based on two-photon polymerization. The alignment printing process consists of three main steps. (1) Coordinate correction between scanning origin (galvo) and writing district (A, B, and C); (2) Loading micro-lens array model and rotation correction (D to J); (3) Starting point alignment and array printing (K).

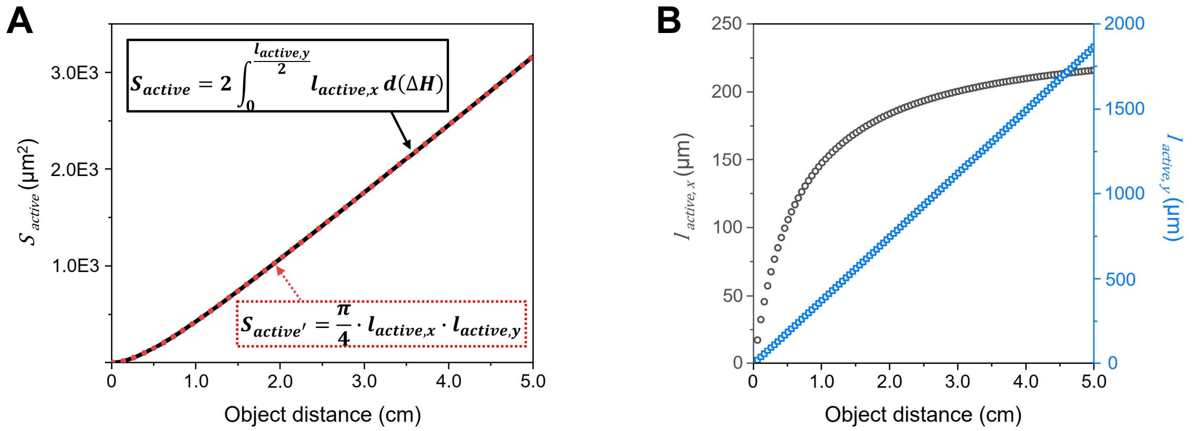

Supplementary Figure 3: Cylindrical compound eye structure model fitting and activation form. Figure A shows that a good model fit is obtained using the elliptic area formula instead of integral calculation. In the region closer to bio-CE, the activation form presented in the x and y directions is different, and the activation length in the x direction is gradually saturated with the increase of the target distance due to the curvature (Figure B).

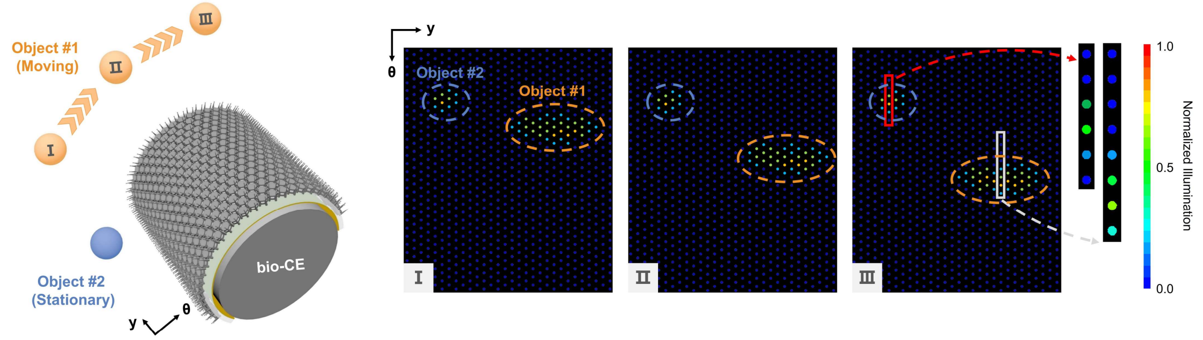

Supplementary Figure 4: Flicker effect in bio-CE imaging. The pixel column affected by the moving target (white column) will switch on and off as the target moves, similar to the flicker effect in the biological compound eye.

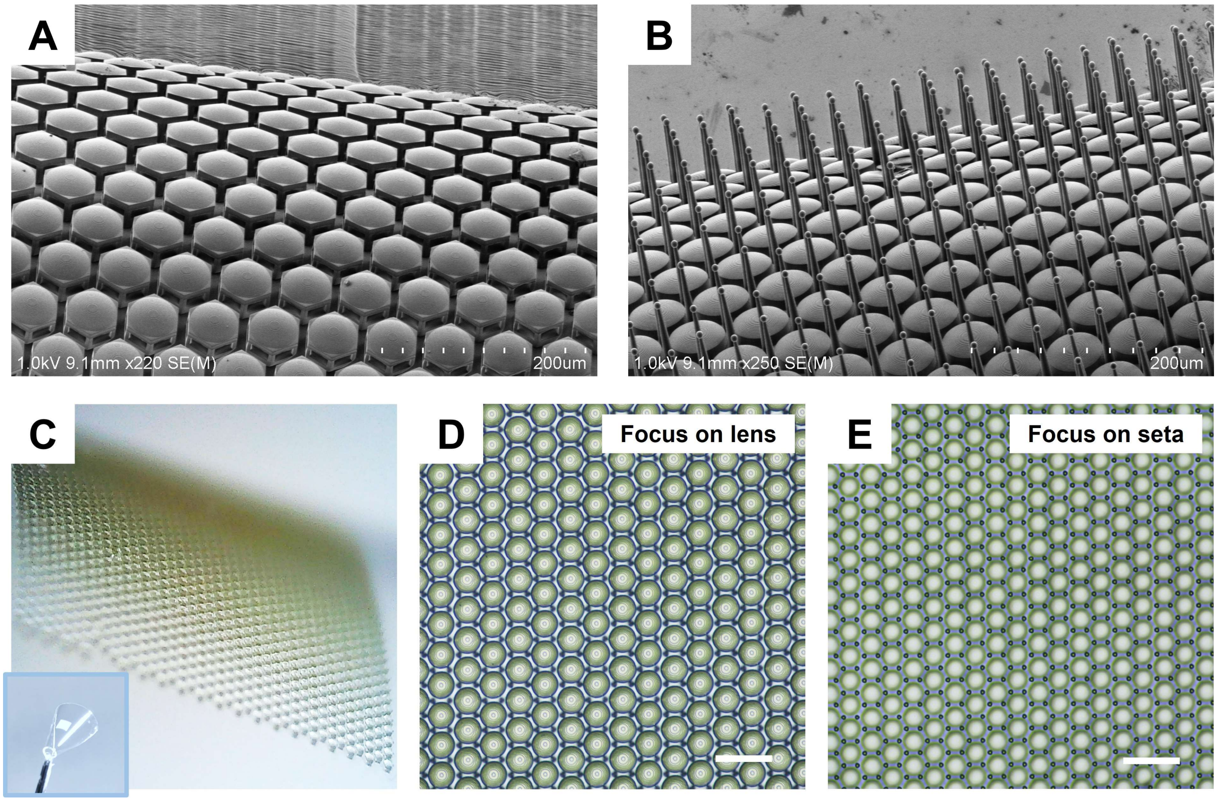

Supplementary Figure 5: Supplement Characterization of different types of micro-lens array. SEM images of a curved micro-lens array and setae-lens array are shown in Figures A and B, respectively. Optical images of a micro-lens array (Figure C) focus on the top of the lens (Figure D) and the top of the setae (Figure E). The scale bar in D and E is 100 µm.

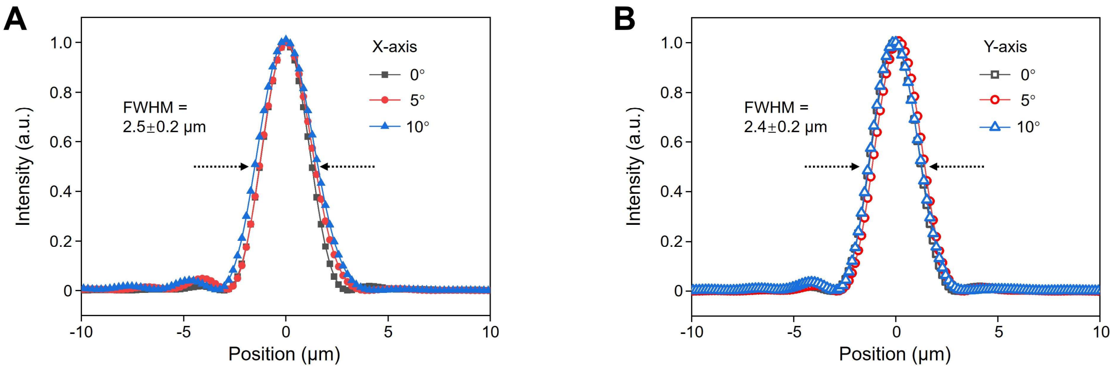

Supplementary Figure 6: Normalized intensity distributions along the X-axis and Y-axis. The full width at half maximum (FWHM) of normalized intensity distribution on the X-axis (Figure A) and Y-axis (Figure B) remains constant under different incident angles, indicating the low aberration in the acceptive angle.

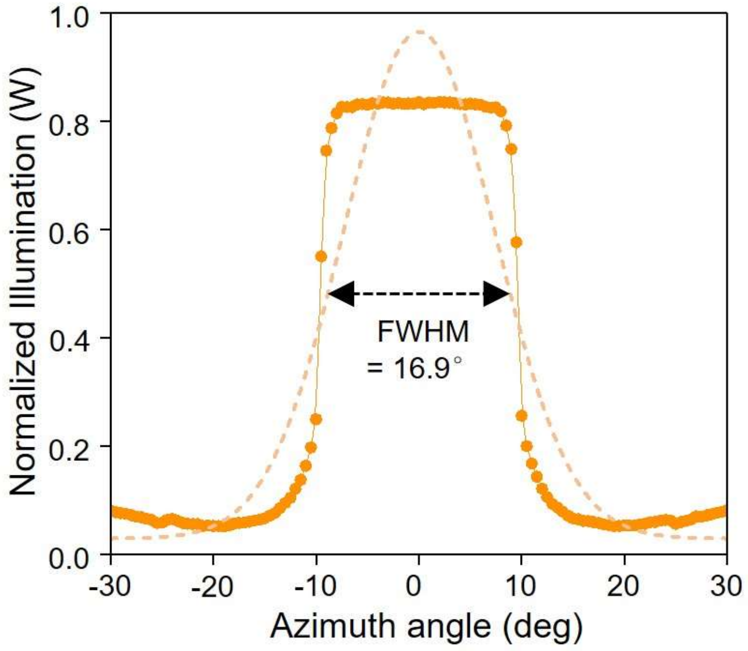

Supplementary Figure 7: The lens receiving angle and the cutting ability of edge rays. The measured receiving angle is flat, with good convergence and stable energy. After leaving the receiving angle, the energy rapidly decreases without scattering peaks. The full width at half maximum (FWHM) after Gaussian fitting represents the size of the receiving angle, which is determined by the combination of lens NA and detector area.

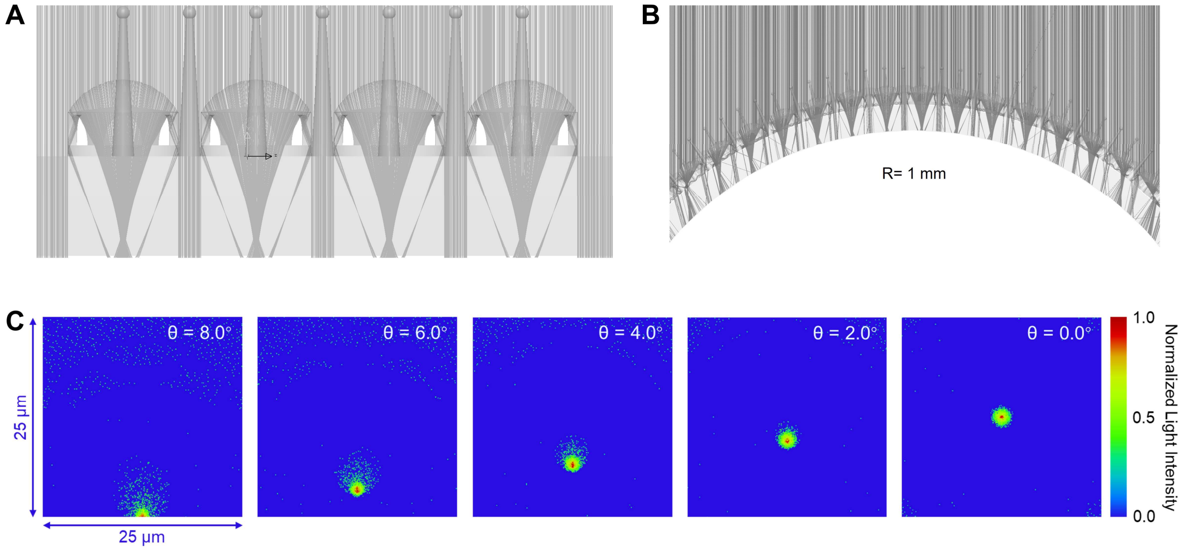

Supplementary Figure 8: Simulation of biomimetic micro lens array ray tracing. As the incident angle gradually increases, the trailing phenomenon formed by the secondary light spot is obvious, but its intensity is much smaller than that of the central main light spot. Therefore, when the incident angle is greater than 6 °, the light spot will detach from the photosensitive surface and cannot cause pixel photoelectric response.

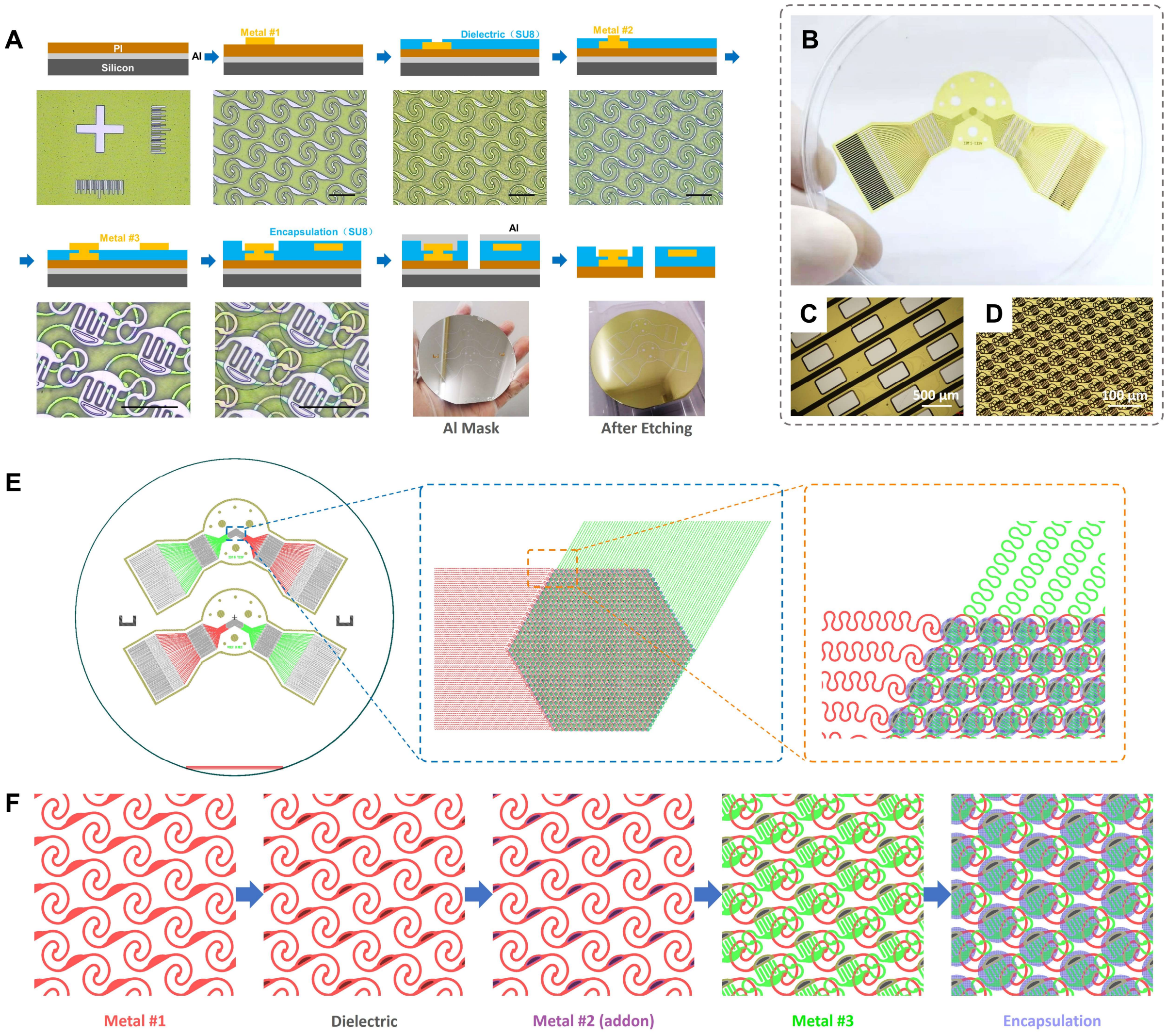

Supplementary Figure 9: The manufacturing details and layout design of the flexible electrode array. A) Processing process 2D schematic diagram and optical pictures of each step, scale bar = 50 μm; B) Device picture released from the silicon wafer; C) The pores of the wiring position design are used to assist the releasing process; D) Zoom in of interdigital electrode array; E) Layout design of bio-CE by L-edit and F) The design details of each layer of mask.

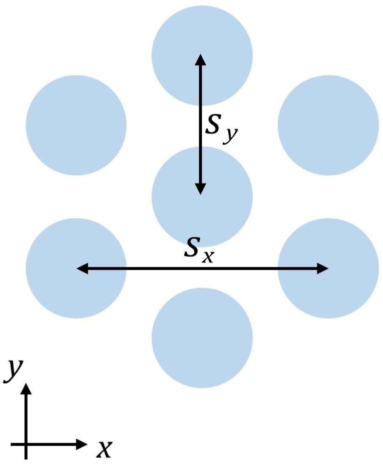

$$FF_{hexagon} = \frac{2\pi R_{lens}^2}{\sqrt{3}S^2}$$

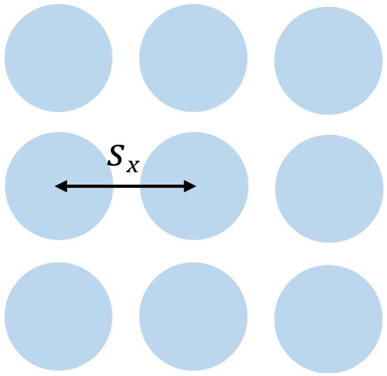

$$FF_{cubic} = \frac{\pi R_{lens}^2}{S^2}$$

$$\frac{R_{lens}}{S} = \frac{5}{12}$$

Supplementary Figure 10: The derivation process of filling coefficient factor. The 120° cross-arrangement configuration achieves a 62.9% fill factor (50 µm pixel diameter, 60 µm inter-pixel spacing), representing an 8.4% improvement over conventional 90° row-column designs (54.5% fill factor).

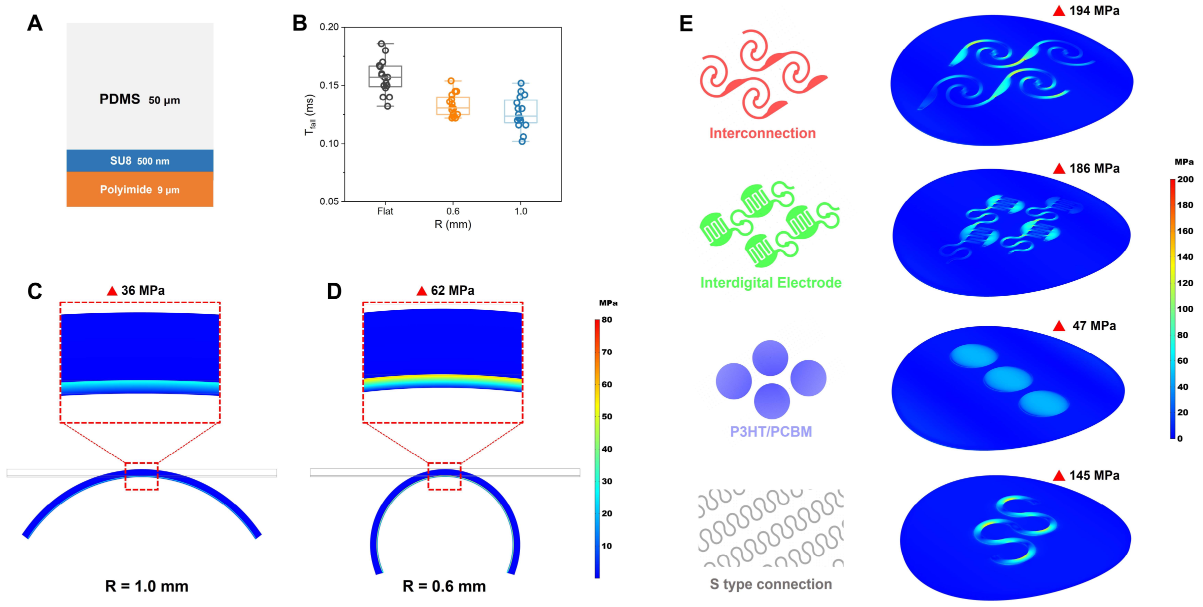

Supplementary Figure 11: Multilayer composite film (PDMS-SU8-Polyimide) bending simulation and effect on light response speed. We use COMSOL Multiphysics to simulate the large deformation process of the composite film (from top to bottom: PDMS 50 µm, SU8 0.5 µm, and Polyimide 9 µm. Figure A). When the curvature radius reaches 1.0 mm (Figure C) and 0.6 mm (Figure D), the maximum stress inside the composite film is located in the interface between PDMS and SU8, which does not exceed the yield stress of SU8 (82 MPa). In the bending process, the pixel's light response speed (mainly restricted by T_{fall}) increases slightly (Figure B). In addition, the stress distribution of the wiring of each layer is shown in Figure E. The mechanical parameters of each material are presented in the Supplementary Table 3.

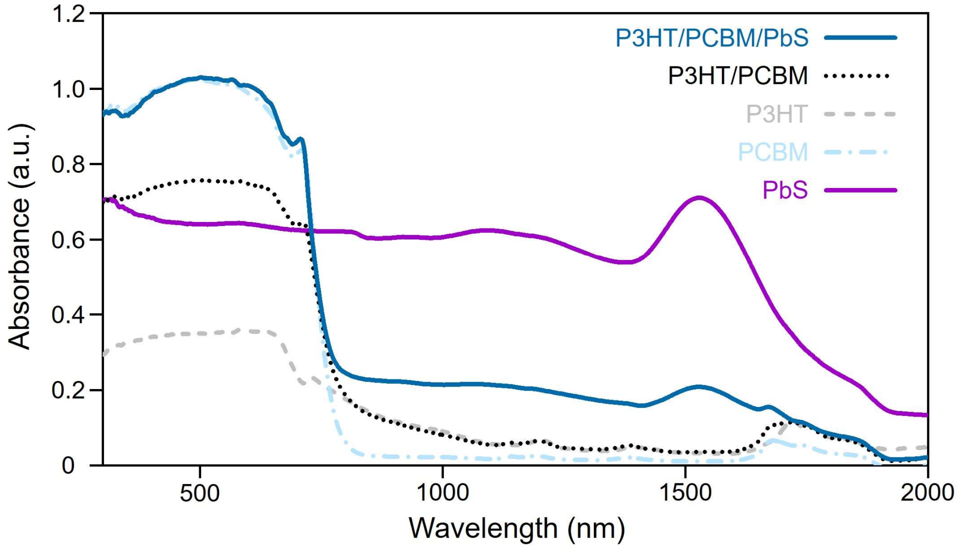

Supplementary Figure 12: Absorption spectra of photoelectric materials. The ultraviolet-visible-near-infrared (UV-VIS-NIR) absorption spectra of each material monomer (P3HT, PCBM, PbS QDs) and composite film material (P3HT/PCBM, P3HT/PCBM/PbS QDs) show that PbS QDs can significantly improve the light absorption of P3HT/PCBM system in the near-infrared wavelength.

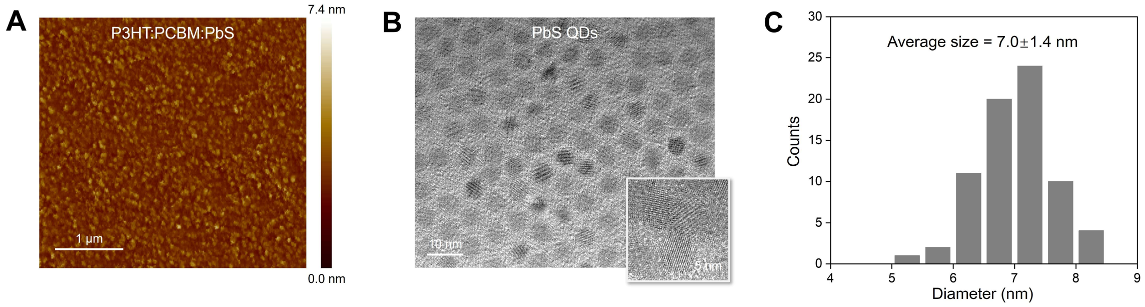

Supplementary Figure 13: Morphology characterization of P3HT/PCBM/PbS QDs organic thin film. The surface morphology of P3HT/PCBM/PbS QDs film scanned by atomic force microscopy (A) and the transmission electron microscope image (B) of PbS quantum dots with an average size of 7.0±1.4 nm(C).

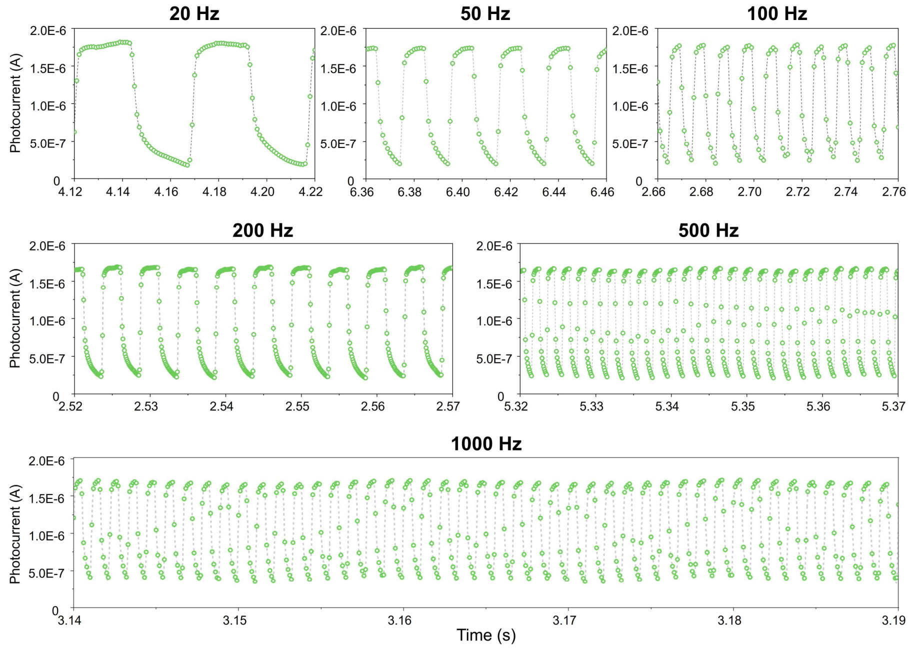

Supplementary Figure 14: Flicker frequency response characteristics of bio-CE. The response characteristics of bio-CE on the high-frequency flicker signal directly affect the detection ability of moving targets. We place a chopper between the laser source (532 nm, 30 W/m^2) and bio-CE to obtain different on-off frequency conditions. The photo response curves of 20 Hz, 50 Hz, and 100 Hz are obtained according to a 1 ms sampling interval and the higher flicker frequency (200 Hz, 500 Hz, and 1000 Hz) under a 0.1 ms sampling interval. As the flickering frequency increase, the device still has excellent switching characteristics (on-off ratio of about 100). The light current maintains stable, but the dark current has increased slightly.

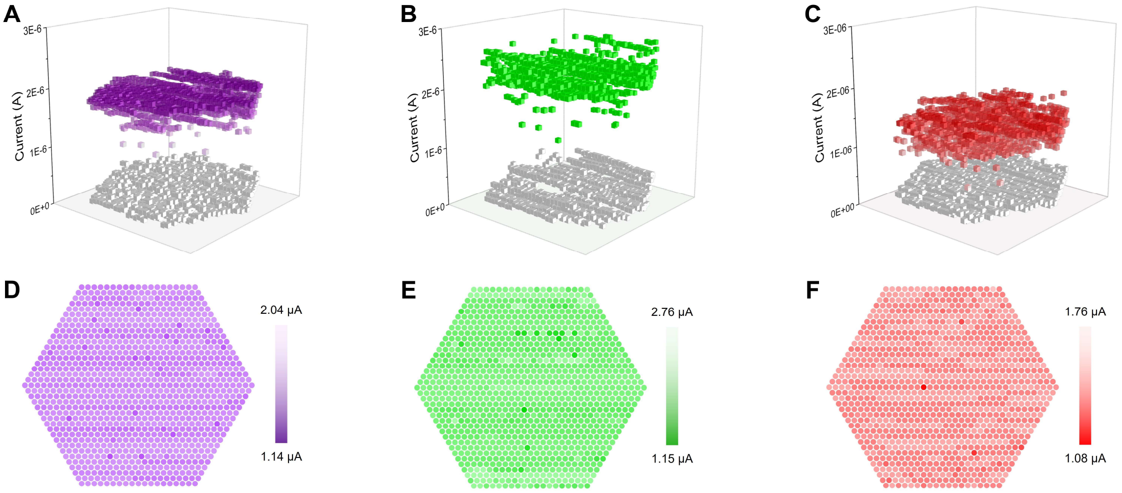

Supplementary Figure 15: Optical response uniformity of all pixels. We perform the optical response uniformity test of bio-CE's 1027 pixels under 365 nm (figure A), 532 nm (figure B), and 1250 nm (figure C) illumination with 45 W/m^2 power density. A reflective beam expander evenly illuminates the entire photodetector array. Corresponding photocurrent distribution is displayed in Figures D, E, and F, respectively. Benefiting from the mixed uniformity of P3HT/PCBM/PbS QDs and the stability of the electrode fabrication process, the optoelectronic performance between pixels is effectively controlled.

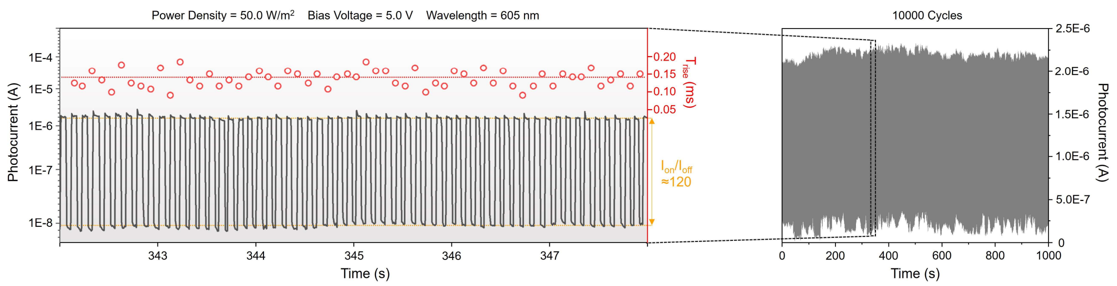

Supplementary Figure 16: Stability of photo response under 10000 cycles. The stability experiment is presented on a single pixel of bio-CE. We place a chopper between the pixel and the light source (605 nm, 50 W/m^2) to obtain a 10 Hz periodic light and dark illumination. During the cycle test, light current and rising time remain stable, and the device does not have apparent degradation or charge accumulation.

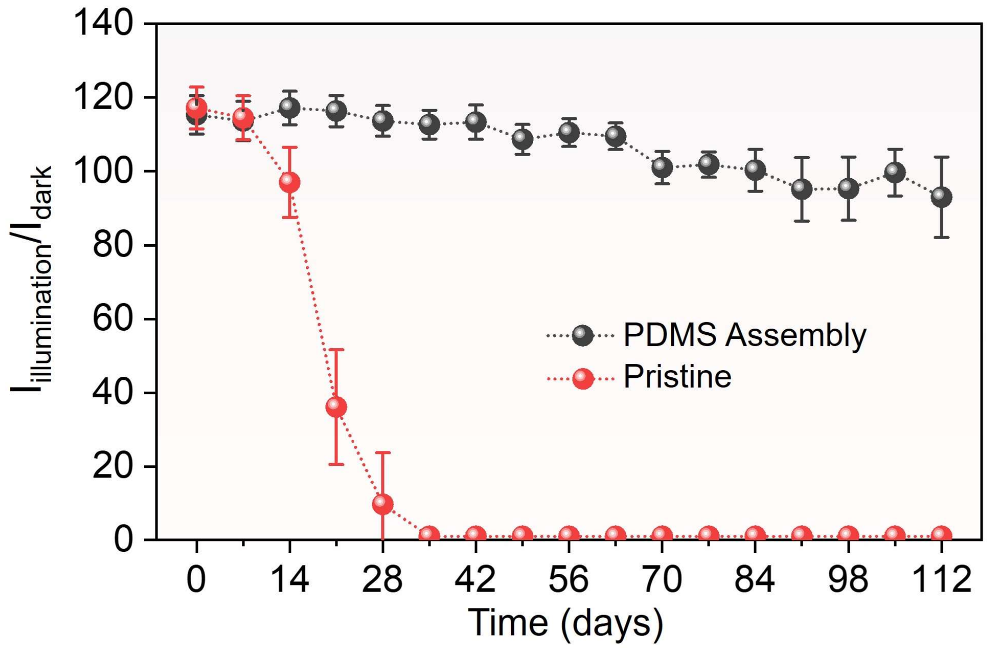

Supplementary Figure 17: Lifetime of bio-CE after PDMS encapsulation. In the bio-CE system, PDMS is used as a flexible optical transmission medium and plays a role in protecting and packaging organic photoelectric detection pixels. The organic semiconductor material (P3HT/PCBM/PbS QDs) exposed to the air rapidly degenerates within two weeks, resulting in the failure of the device (red). The pixels with PDMS packaging can avoid the erosion of oxygen and humidity, which maintain stability within a few months (gray).

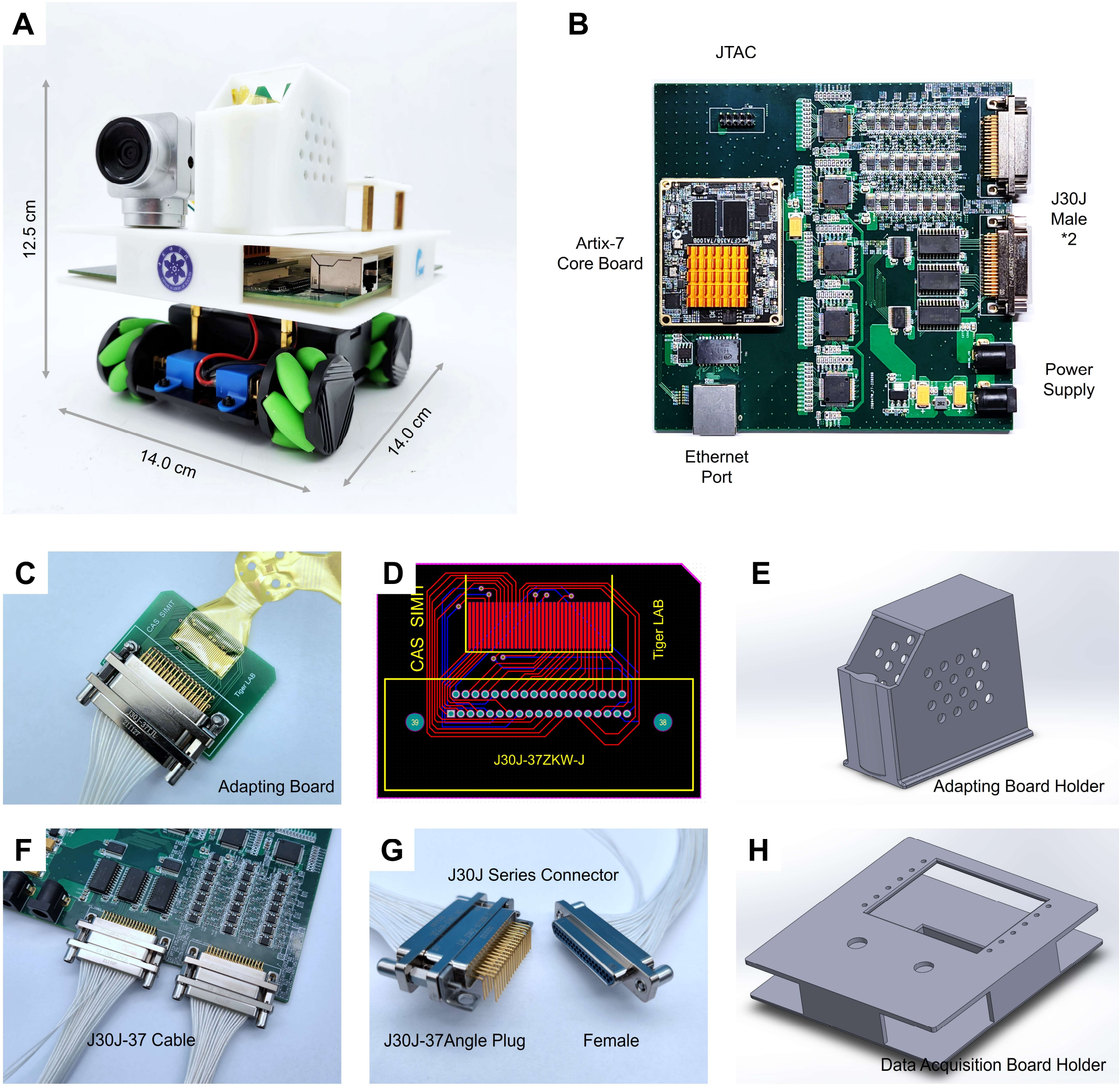

Supplementary Figure 18: Details and connections of the bio-CE-based unmanned vehicle platform. The bio-CE applied unmanned vehicle consists of a Mecanum wheels chassis, data acquisition board, and bio-CE/CCD camera from bottom to top. The overall dimensions are shown in Figure A. The data acquisition board (Figure B) is placed into a customized 3D-printed holder (Figure H), connected to the adapting board (ACF bonding with bio-CE, Figure C, D) by the J30J connector (Figure F, G). The acquisition and processing of photoelectric response, power supply, and motion control of chassis are all completed by the Artix-7 FPGA-based data acquisition board.

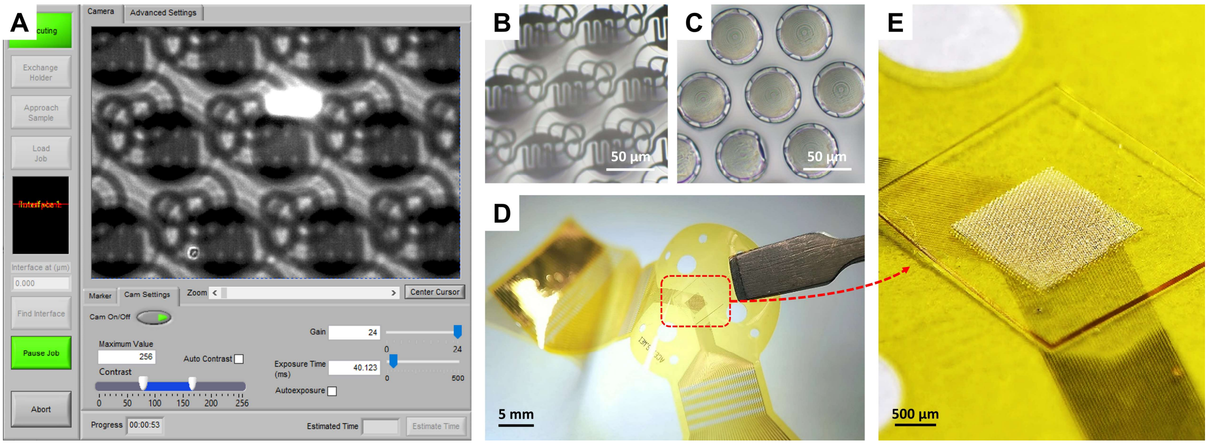

Supplementary Figure 19: Alignment printing process and results. After the maker alignment and double-checking of the lens model, we can load the transferred model (.job) into the Nanowrite software. Besides the size information of the lens model, the .job file also contains parameters such as scanning speed, area size and direction, and processing laser intensity. Figure A shows the processing software interface of the Nanowrite. Figure B and C are optical images of the fabricated alignment lens sample, which focus on the pixel plane and top of the lens, respectively. Full frame fabrication results are presented in Figures D and E.

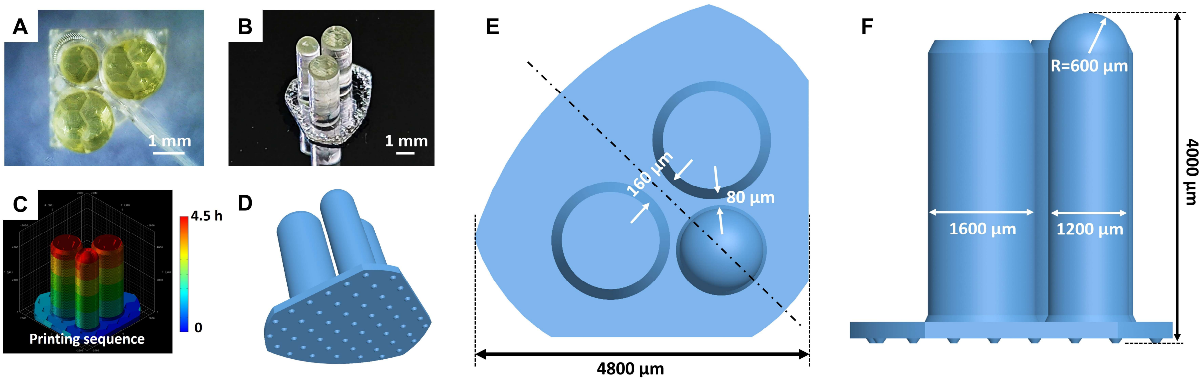

Supplementary Figure 20: Fixed curvature holder of bio-CE. The bio-CE is applied to a cylindrical holder to acquire destinated curvature. We can avoid loosening by adjusting the pillar center distance to clamp the device. A rightly fixed device shown in figure A, this miniature holder (Figure B) is fabricated by two-photon polymerization with the 3D Microfabrication Solution Set Large Features (3D LF), representing a fast fabrication process for millimeter models. The total printing time of the 0.6 mm radius holder is about 4.5 hours (Figure C). Besides, to separate the mold from the printing base by heat gradient, we have designed arrays of the round platform at the bottom of the mold (Figure D). Figures E and F show detailed parameters of the fixed curvature holder.

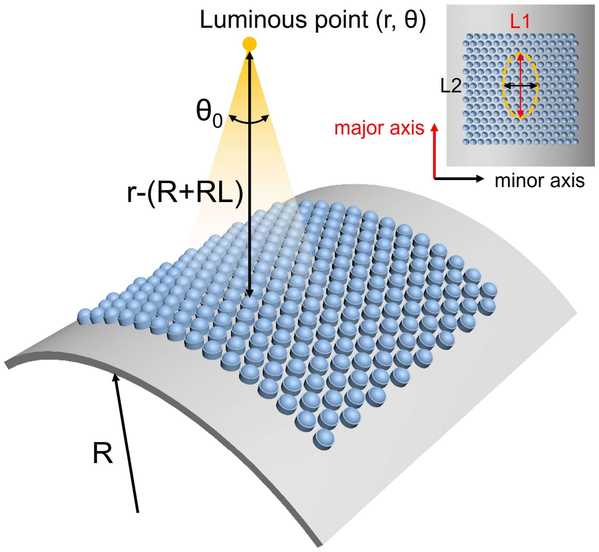

$$L1 = 2\,tan\left(\frac{\theta_0}{2}\right)\{r-(R+RL)\}$$

$$L2 = \left\{2(R+RL) - \frac{2(R+RL)^2}{r}\right\} tan\left(\frac{\theta_0}{2}\right)$$

$$n = \left[\frac{\pi tan^2\left(\frac{\theta_0}{2}\right)(R+RL)}{\frac{\sqrt{3}}{2}S^2} \times \frac{\{r-(R+RL)\}^2}{r}\right]$$

Supplementary Figure 21: The bio-CE imaging model. We establish the bio-CE imaging model based on the illumination angle of the light source and the target distance, where S is the distance between the micro lenses.

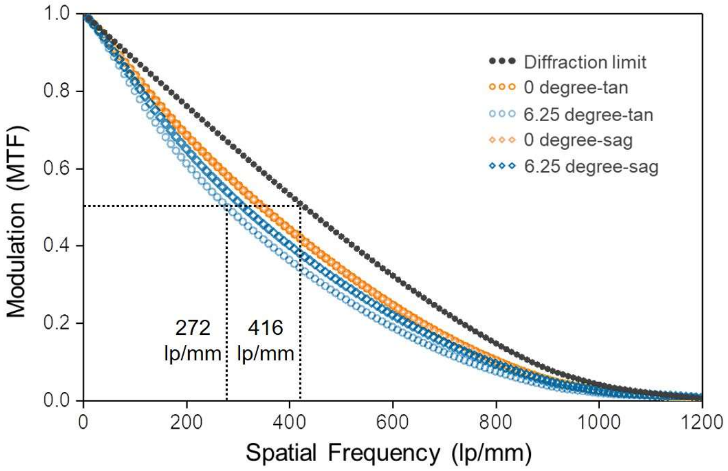

Supplementary Figure 22: Resolution of the fabricated microlens array. MTF represents the resolution of the fabricated microlens array. Observe a specific target card using a lens and read the MTF value, with 0.5 being the resolution contrast optimization point. The maximum resolution obtained from simulation is 416 lp/mm (2.4 μm), while the measured resolution is 3.7 μm, which is better than the 70 μm of the human eye.

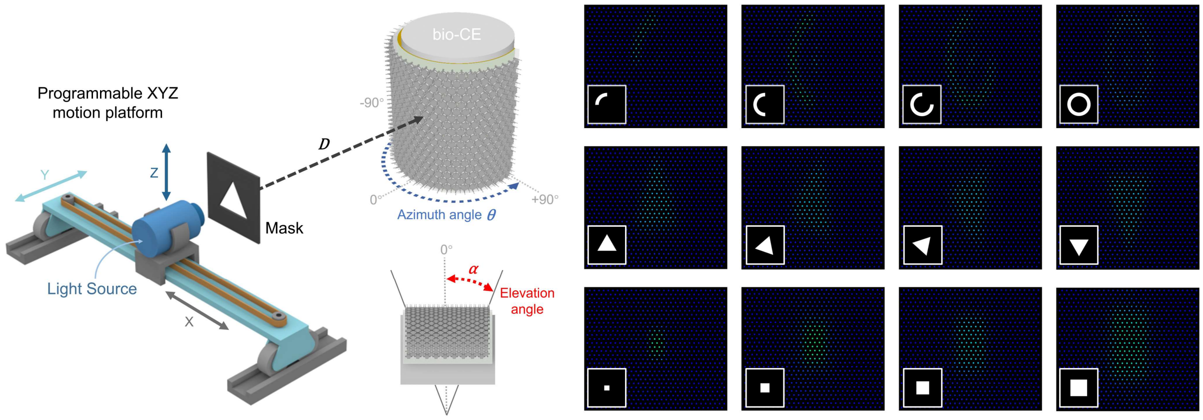

Supplementary Figure 23: Imaging of different shapes and movements by bio-CE. By switching different masks to achieve shape changes, rotation, scaling, and other movements, although there is a certain degree of shape distortion in the imaging results, the single-frame imaging results show that bio-CE is very sensitive to the shape changes of targets.

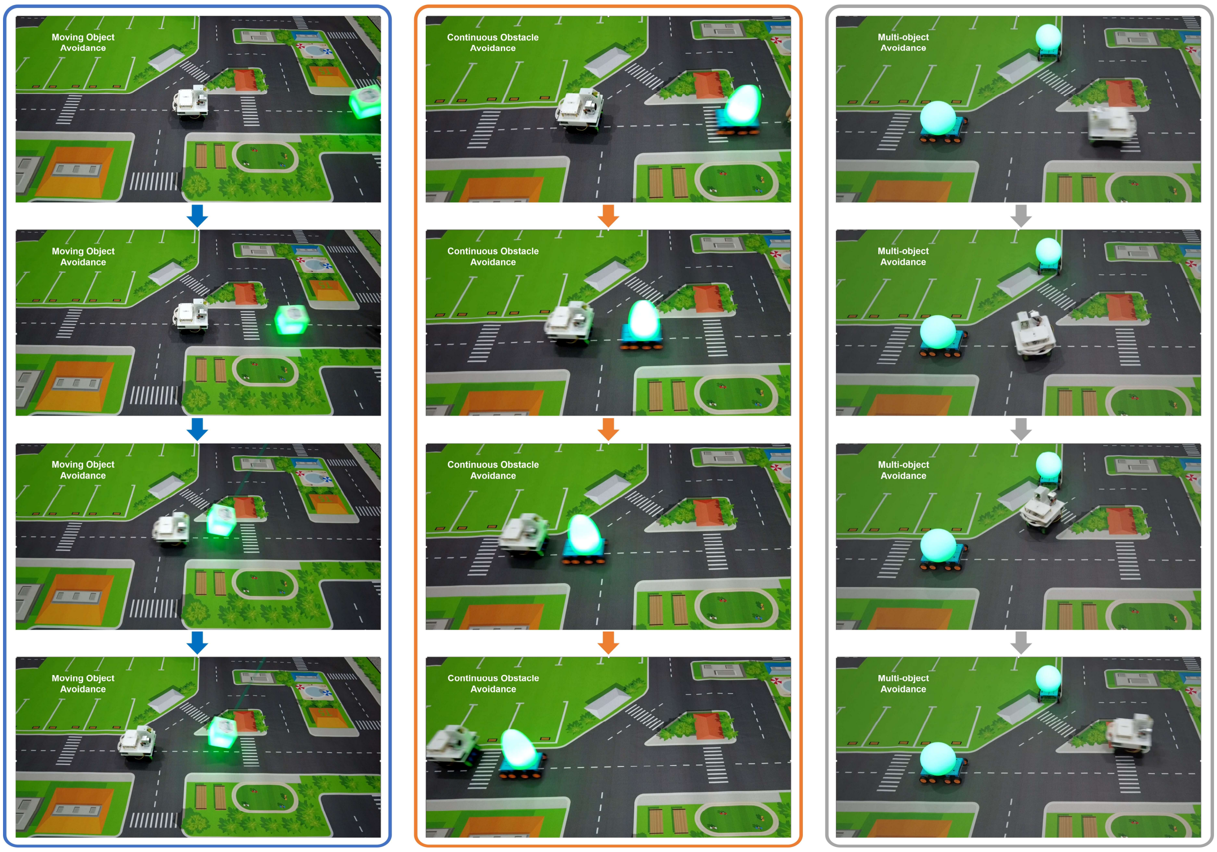

Supplementary Figure 24: Test results of bio-CE microsystem in dynamic environments with multiple moving obstacles. The scenario design incorporates both stationary and dynamic obstacles. Under these configurations, the active and passive obstacle avoidance performance of the bio-CE microsystem was systematically evaluated and analyzed.

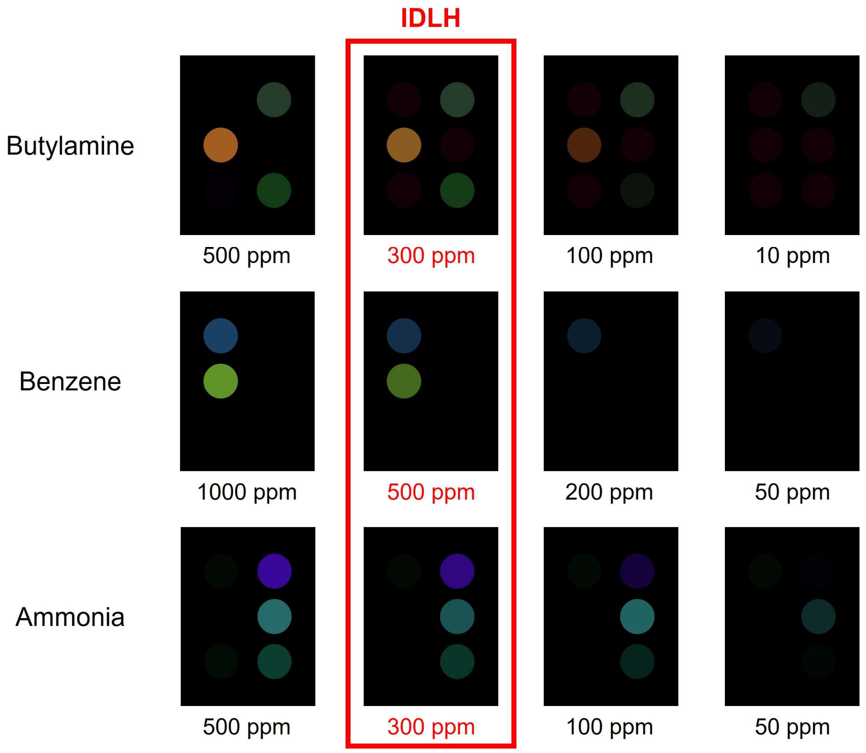

Supplementary Figure 25: Colorimetric response under different concentration. The responses of colorimetric arrays to different concentrations of butylamine, benzene, and ammonia are shown from top to bottom accordingly. The colorimetric array presents a significant and stable response at IDLH concentration, and the response gradually weakens with the decrease of the target concentration.

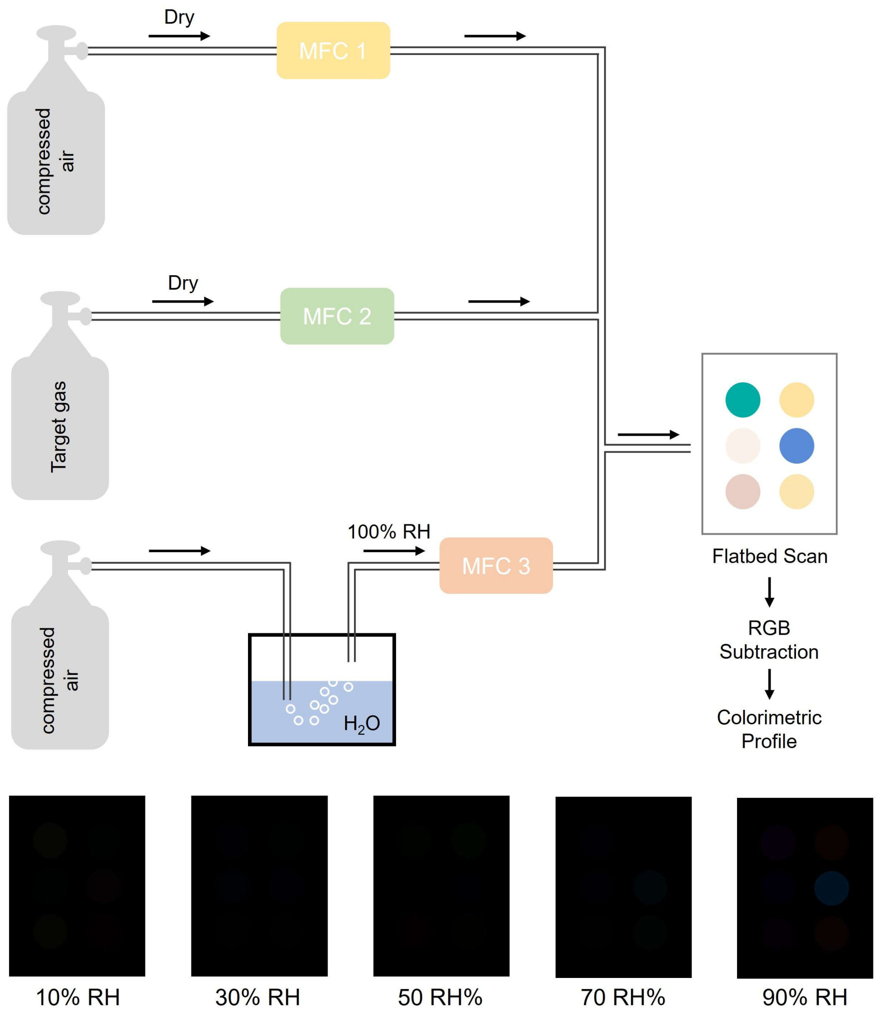

Supplementary Figure 26: Humidity stability of colorimetric array. We designed and built a valve system to verify the colorimetric array response of humidity. Similar methods were used to test target gases of different concentrations. The results show that introducing water vapor has a negligible effect on the color pattern.

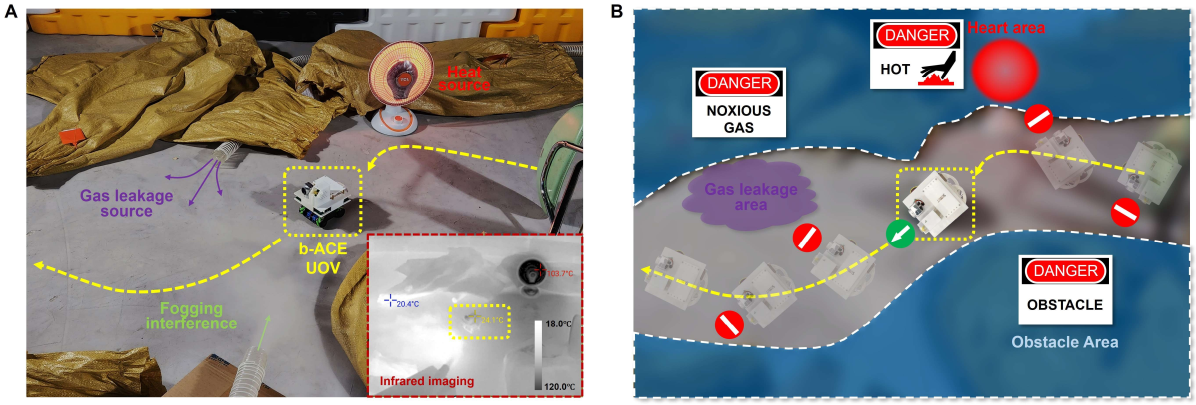

Supplementary Figure 27: Object attribute recognition experiment. Target recognition and autonomous obstacle avoidance testing of simulated scenes by unmanned vehicles equipped with biomimetic compound eye systems in natural light environments.

Supplementary Table 1. Comparison of design parameters and performance of artificial compound eyes

	System overall parameters				Lens array parameters						Photoreceptor parameters				
	Layout type	Size (mm)	Thickness (mm)	FOV (°)	Ommatidia number	Fill factor	Acceptive angle (°)	Interommatidial angle (°)	Angular sensitivity function (°)	F number	Lens diameter (mm)	Receptor number	Receptor diameter (μm)	On-off ratio	Frame rate(fps)
Biomimetic artificial compound eye [1]	Curved micro-lens array integrated with flat photoreceptor array/CMOS sensor	D=2.5	0.3	—	8370	—	—	1.5	1.1~4.4	1.93	0.025	transmission confocal microscope (Zeiss 510, Carl Zeiss MicroImaging, Incorporated, Thornwood, NY)			
BAC-eye [2]		D=5	—	360*170	522	—	—	—	1.9	—	0.18	CMOS camera (EO-18112, Edmund Optics Inc., USA)			
Dragonfly-Eye-Inspired Artificial Compound Eye [3]		D=5	—	360*132.8	30000	100%	—	0.57	5.8	—	0.025	Commercial CCD camera			
BIC eye [4]		D=0.32	—	360*97	150	100%	—	7.4	1.8	1.25	0.008	Commercial CCD camera			
μ-CE [5]		D=0.4	—	360*90	19~160	100%	—	—	12.1	—	0.04	Commercial CMOS detector (OV9734, OmniVision Company)			
Artificial RSCE [6]	Curved micro-lens array integrated with curved photodetector array	D=31	0.4	360*165	168100	—	—	—	—	—	0.08	—	—	—	—
Convex Origami electronic eye [7]		D=4.5	—	—	676	—	60	—	—	—	0.13	676	113	$\sim 10^2$	—
Hemispherical apposition camera [8]		D=15	0.95	360*160	256	59.4%	9.7	11.0	—	—	0.8	256	160	$<10^2$	—
CurvACE [9]		D=12.8	~1	180*60	630	45.8%	4.2	~4.2	3.7~4.2	2.4	0.172	630	20	—	1.5k
This work		D=1.2	0.085	180*60	1047	62.9%	16.9	4.86	4.20	4.25	0.052	1047	20	$\sim 10^2$	1.0k

Supplementary Table 2：Comparison of the performance of bio-CE with different types of biological eyes.

Reference	Layout type	Focal length (mm)	Lens diameter (mm)	FOV (°)	F number	Acceptive angle (°)	Sensitivity (W/m^2)	Dynamic range (dB)	Spectral range (nm)	Critical flicker fusion frequency (Hz)
Human [10]	Single aperture	23	7	15~30	3.3	0.007	0.23	~100	380-700	30~55
Fishes [11] [12]	Single aperture	5.839	4.79	~150	—	~150	—	~70	550-910	40-80
Bee [10] [13] [14]	Apposition compound eye	0.06	0.025	150*360	2.4	1.9	0.24	—	300-650	~300
Moth [10] [15] [16]	Refractive superposition eye	0.17	0.4	140*360	0.4	13	218	~120	300-600	100-140
Bibio Marci [10][17]	Neural superposition eye	0.07	0.021	140*360	0.6	2.0	0.27	—	350~600	~250
This work	Apposition compound eye	0.085	0.052	180*60	4.25	16.9	0.67	~100	350~1550	1k~5k

Supplementary Table 3：Material parameters for flexible electrode bending simulation.

Materials	Young's modulus (MPa)	Poisson's ratio	Density (kg/m^3)	Yield stress (MPa)	Reference
PDMS	C_{10} = 0.662<br>D_{10}=0.25	0.5	970	2.24	18-26
SU8	2920	0.22	1200	82	
Polyimide	3140	0.34	1420	112	
Au film（500 nm）	5300	0.43	19320	200-240	
P3HT:PC[60]BM	1970	0.35	1280	71	

Supplementary Table 4: The critical concentration and basic properties of target gases.

Name	Category	Formula	SVP (mmHg)	IDLH(ppm)	PELs(ppm)	UEL(%VOL)	LEL(%VOL)
Triethylamine	Amine	$C_6H_{15}N$	54.0 (25°C)	200	25	8.0	1.2
Butylamine		$C_4H_{11}N$	97.3 (25°C)	300	5	9.8	1.7
octylamine		$C_8H_{19}N$	0.941 (20°C)	300	-	8.2	1.6
Benzene	VOCs	C_6H_6	100.9 (20°C)	500	5	7.8	1.2
Methanol		CH_3OH	410 (50°C)	6000	200	36	6
THF	TICs	C_4H_8O	145 (37°C)	2000	200	11.8	1.8
Hydrogen Chloride		HCl	-	50	5	-	-
Nitric Acid		HNO_3	-	25	2	-	-
Ammonia		NH_3	-	300	50	28	15
Carbon monoxide	Combustible Gas	CO	-	1200	50	74	12.5

Reference

[1] Jeong, K. H., Kim, J. & Lee, L. P. Biologically inspired artificial compound eyes. *Science* 312, 557-561 (2006).

[2] Dai, B. et al. Biomimetic apposition compound eye fabricated using microfluidic-assisted 3D printing. *Nat Commun* 12, 6458 (2021).

[3] Deng, Z. et al. Dragonfly-Eye-Inspired Artificial Compound Eyes with Sophisticated Imaging. *Adv. Funct. Mater.* 26, 1995-2001 (2016).

[4] Wu, D. et al. Bioinspired Fabrication of High-Quality 3D Artificial Compound Eyes by Voxel-Modulation Femtosecond Laser Writing for Distortion-Free Wide-Field-of-View Imaging. *Advanced Optical Materials* 2, 751-758 (2014).

[5] Hu, Z. Y. et al. Miniature optoelectronic compound eye camera. *Nat Commun* 13, 5634 (2022).

[6] Huang, C. C. et al. Large-field-of-view wide-spectrum artificial reflecting superposition compound eyes. *Small* 10, 3050-3057 (2014).

[7] Zhang, K. et al. Origami silicon optoelectronics for hemispherical electronic eye systems. *Nat Commun* 8, 1782 (2017).

[8] Song, Y. M. et al. Digital cameras with designs inspired by the arthropod eye. *Nature* 497, 95-99 (2013).

[9] Floreano, D. et al. Miniature curved artificial compound eyes. *Proc Natl Acad Sci U S A* 110, 9267-9272 (2013).

[10] Land, M. F. The optics of animal eyes. *Contemporary Physics* 29, 435-455 (1988).

[11] Jagger, W. S. & Sands, P. J. A wide-angle gradient index optical model of the crystalline lens and eye of the rainbow trout. *Vision Res.* 36, 2623-2639 (1996).

[12] Mass, A. M. & Supin, A. Y. Adaptive features of aquatic mammals' eye. *Anat. Rec.* 290, 701-715 (2007).

[13] Goldsmith, T. H. Optimization, constraint, and history in the evolution of eyes. *Q. Rev. Biol.* 65, 281-322 (1990).

[14] Peitsch, D. et al. The spectral input systems of hymenopteran insects and their receptor-based color-vision. *J. Comp. Physiol. A-Sens. Neural Behav. Physiol.* 170, 23-40 (1992).

[15] Nordtug, T. Dynamics and sensitivity of the pupillary system in the eyes of noctuid moths. *J. Insect Physiol.* 36, 893-901 (1990).

[16] Horridge, G. A., Marcelja, L. & Jahnke, R. Retinula cell responses in a moth superposition eye. *Proceedings of the Royal Society Series B-Biological Sciences* 220, 47-+ (1983).

[17] Borst, A. Drosophila's View on Insect Vision. *Curr. Biol.* 19, R36-R47 (2009).

[18] Mendez-Hernandez, J. M., Hernandez-Perez, A., Oviedo-Mendoza, M. & Hernandez-Rodriguez, E. Effects of mechanical deformations on P3HT: PCBM layers for flexible solar cells. *Mech. Mater.* 154, 8 (2021).

[19] Savagatrup, S., Makaram, A. S., Burke, D. J. & Lipomi, D. J. Mechanical Properties of Conjugated Polymers and Polymer-Fullerene Composites as a Function of Molecular Structure. *Adv. Funct. Mater.* 24, 1169-1181 (2014).

[20] Chang, W.-Y., Fang, T.-H. & Lin, Y.-C. Physical characteristics of polyimide films for flexible sensors. *Applied Physics A* 92, 693-701 (2008).

[21] Xu, T. G. et al. Characterization of the mechanical behavior of SU-8 at microscale by viscoelastic analysis. *J. Micromech. Microeng.* 26, 12 (2016).

[22] Chasiotis, I. et al. Strain rate effects on the mechanical behavior of nanocrystalline Au films. *Thin Solid Films* 515, 3183-3189 (2007).

[23] Hasenkamp, W. et al. Polyimide/SU-8 catheter-tip MEMS gauge pressure sensor. *Biomed. Microdevices* 14, 819-828 (2012).

[24] Lee, S. J. et al. Measurement of Young's modulus and Poisson's ratio for thin Au films using a visual image tracing system. *Current Applied Physics* 9, 75-78 (2009).

[25] Robin, C. J., Vishnoi, A. & Jonnalagadda, K. N. Mechanical Behavior and Anisotropy of Spin-Coated SU-8 Thin Films for MEMS. *J. Microelectromech. Syst.* 23, 168-180 (2014).

[26] Savagatrup, S. et al. Mechanical degradation and stability of organic solar cells: molecular and microstructural determinants. *Energy Environ. Sci.* 8, 55-80 (2015).

[27] Lee, M. et al. An amphibious artificial vision system with a panoramic visual field. *Nature Electronics* 5, 452-459 (2022).
