# Supplementary material for: An insect-scale artificial visual-olfactory bionic compound eye
Source: Nat Commun. 2026 Feb 2;17:2259. doi: 10.1038/s41467-026-68940-0 (PMC12966333; doi:10.1038/s41467-026-68940-0)
Supplement: Supplementary file 2 — Description of Additional Supplementary Files [file 41467_2026_68940_MOESM2_ESM.pdf]

Description of Additional Supplementary Files

Supplementary Movie 1: Anti-fogging Properties of Drosophila Compound Eye and Artificial Seta-lens Structure

Supplementary Movie 2: Demonstration of obstacle avoidance function of bio-CE based omnidirectional unmanned vehicle
